# Medicinal plants used for treatment of malaria by indigenous communities of Tororo District, Eastern Uganda

**DOI:** 10.1186/s41182-023-00526-8

**Published:** 2023-06-12

**Authors:** John R. S. Tabuti, Samuel Baker Obakiro, Alice Nabatanzi, Godwin Anywar, Cissy Nambejja, Michael R. Mutyaba, Timothy Omara, Paul Waako

**Affiliations:** 1 Department of Environmental Management, Makerere University, P.O. Box 7062, Kampala, Uganda; 2 Department of Pharmacology and Therapeutics, Faculty of Health Sciences, Busitema University, P.O. Box 1460, Mbale, Uganda; 3 Department of Plant Sciences, Microbiology & Biotechnology, College of Natural Sciences, Makerere University, P.O. Box 7062, Kampala, Uganda; 4 Natural Chemotherapeutics Research Institute (NCRI), Ministry of Health, P.O. Box 4864, Kampala, Uganda; 5 National Drug Authority, Ministry of Health, P.O. Box 23096, Kampala, Uganda; 6 Institute of Chemistry of Renewable Resources, Department of Chemistry, University of Natural Resources and Life Sciences, Vienna (BOKU), The Tulln University and Research Center (UFT), Konrad-Lorenz-Straße 24, 3430 Tulln an der Donau, Austria

**Keywords:** Antimalarial resistance, Ethnobotany, Indigenous knowledge, Malaria, Medicinal plants, Traditional medicine

## Abstract

**Background:**

Malaria remains the leading cause of death in sub-Saharan Africa. Although recent developments such as malaria vaccine trials inspire optimism, the search for novel antimalarial drugs is urgently needed to control the mounting resistance of *Plasmodium* species to the available therapies. The present study was conducted to document ethnobotanical knowledge on the plants used to treat symptoms of malaria in Tororo district, a malaria-endemic region of Eastern Uganda.

**Methods:**

An ethnobotanical study was carried out between February 2020 and September 2020 in 12 randomly selected villages of Tororo district. In total, 151 respondents (21 herbalists and 130 non-herbalists) were selected using multistage random sampling method. Their awareness of malaria, treatment-seeking behaviour and herbal treatment practices were obtained using semi-structured questionnaires and focus group discussions. Data were analysed using descriptive statistics, paired comparison, preference ranking and informant consensus factor.

**Results:**

A total of 45 plant species belonging to 26 families and 44 genera were used in the preparation of herbal medicines for management of malaria and its symptoms. The most frequently mentioned plant species were *Vernonia amygdalina*, *Chamaecrista nigricans*, *Aloe nobilis*, *Warburgia ugandensis*, *Abrus precatorius*, *Kedrostis foetidissima*, *Senna occidentalis*, *Azadirachta indica* and *Mangifera indica*. Leaves (67.3%) were the most used plant part while maceration (56%) was the major method of herbal remedy preparation. Oral route was the predominant mode of administration with inconsistencies in the posology prescribed.

**Conclusion:**

This study showed that the identified medicinal plants in Tororo district, Uganda, are potential sources of new antimalarial drugs. This provides a basis for investigating the antimalarial efficacy, phytochemistry and toxicity of the unstudied species with high percentage use values to validate their use in the management of malaria.

**Supplementary Information:**

The online version contains supplementary material available at 10.1186/s41182-023-00526-8.

## Background

Malaria remains one of the diseases with the highest human morbidities and mortalities in the world [1]. It is one of the greatest obstacles to socio-economic development, especially in the developing countries where it is endemic [2, 3]. In 2019, there were 229 million global malaria cases with a case incidence of 57% [1]. The African continent accounted for 95% of all global malaria cases, with Uganda accounting for 5% of these. In the same year, the global malaria mortality rate stood at 10 persons per 100,000 at risk. In 2020, there was a marked increase in global malaria incidence with about 14 million more cases and 69,000 additional deaths compared to 2019. About two-thirds of the mortalities were attributed to disruptions in malaria services during the COVID-19 pandemic, particularly in countries of the WHO African Region [1].

In East Africa, malaria remains endemic in the Lake Victoria basin, with *Anopheles gambiae* and *Anopheles funestus* being the implicated vectors perpetuating it [4, 5]. *Plasmodium falciparum* and *Plasmodium vivax* are the two deadliest malarial parasites in sub-Saharan Africa. Currently, artemisinin-based combination therapies (ACT) are the treatment of choice for malaria [6–8]. They are available for free but are sometimes hard to access in Ugandan government health centres and hospitals [9]. Early diagnosis and prompt treatment of malaria should occur within 24 h of the onset of symptoms to decrease the risk of severe complications and onward transmission which occurs within a few hours for *P. falciparum* malaria [10, 11]. Unfortunately, there are delays in seeking care, obtaining a diagnosis and receiving appropriate treatment by Ugandans which is associated with fatal malaria. While tremendous progress has been made in the fight against malaria through the improvement of health system performance and increased public knowledge about the disease, increasing resistance to commonly used treatments (including ACT) is presenting new challenges to malaria control and eradication programmes [12, 13]. Therefore, malaria cases remain high in Uganda, despite the availability of ACT [14, 15]. Due to the high risk of morbidity and mortality, the Ugandan government spends a lot of money on procuring antimalarial drugs for its citizens.

In spite of the success achieved regarding universal health coverage, traditional and complementary medicines have not remained an integral component of the health care system of Uganda. Traditional medicine is culturally accepted, readily available, free or cheap and is perceived to be safe and efficacious. The evaluation of plant materials for new drugs is justified because many modern allopathic medicines including antimalarial drugs originated from plants [16]. For example, the two main groups (artemisinin and quinine derivatives) of modern antimalarial drugs contain lead compounds derived from *Cinchona* species and *Artemisia annua* plant extracts, respectively [17]. Uganda forms part of the East African botanical plate which is rich in medicinal plants. Communities in different regions of the country use different herbs within their geographical range, although a few common herbs are used by different communities across the country [18]. However, ethnobotanical documentation of the medicinal plants used to treat malaria is far from complete among various communities in the country. Therefore, this study aimed at generating information that will contribute to the development of efficacious and safe antimalarial drugs, by documenting and prioritizing plants used for treating malaria in Tororo district, Eastern Uganda.

## Methods

### Study area

**Fig. 1:**
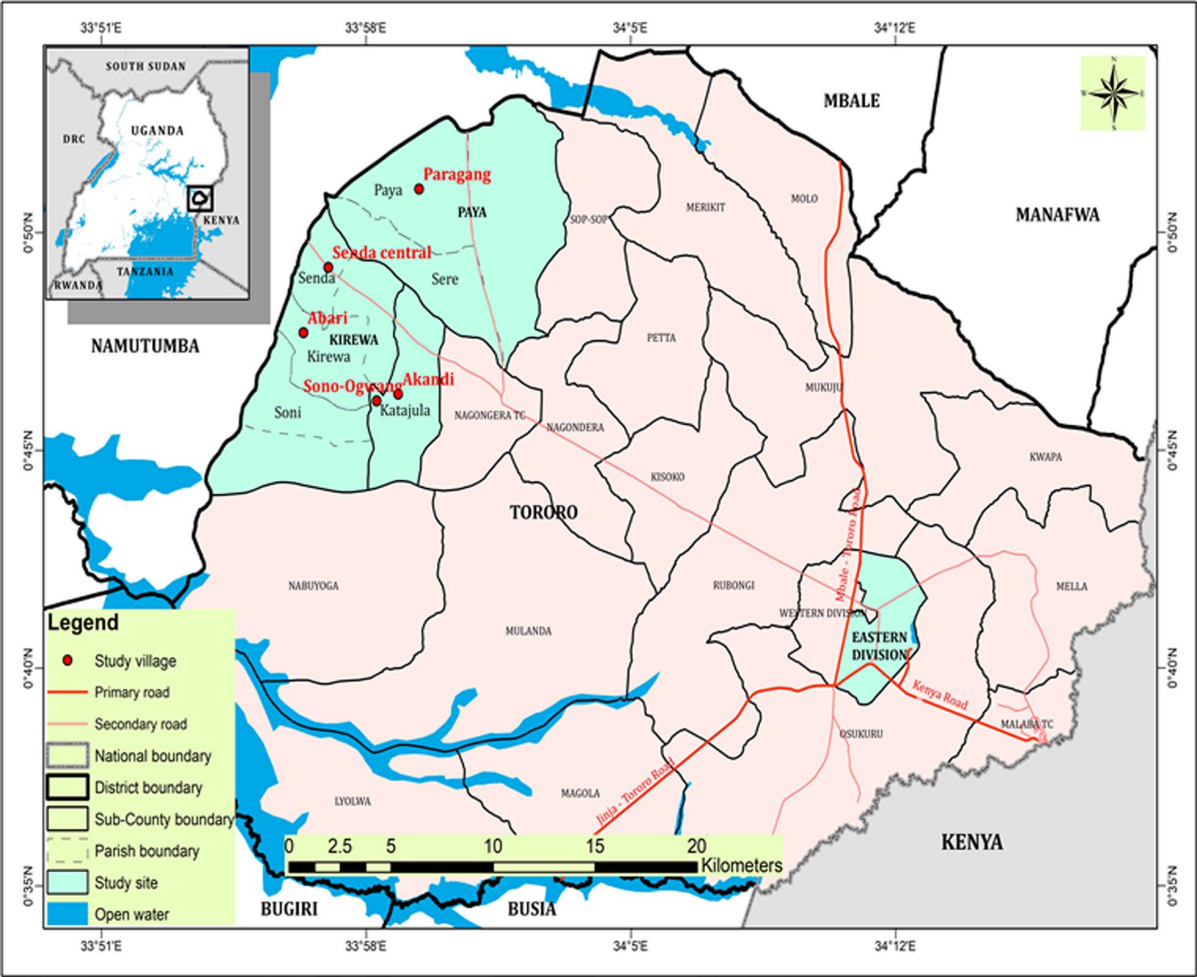
Map showing the location of the study area. Inset is the map of Uganda showing the location of Tororo district

This was an ethnobotanical survey conducted in Tororo district (0° 41′ 34.0008″ N and 34° 10′ 51.9960″ E), Eastern Uganda (Fig. 1). Tororo borders Bugiri district to the West, Butaleja district to the North, Busia district to the South, Republic of Kenya to the East and Mbale district to the North East. It has a population of about 597,500 people distributed as 51.2% females, and 48.8% males. The majority of the people (86%) live in the rural areas. The major economic activity in the area is subsistence farming. Tororo district is one of the malaria-endemic districts with an entomological infective rate of 591, making it one of the most malaria burdened districts in Uganda [19, 20]. Despite government efforts to increase access to health services from health facilities, residents of Tororo district still rely on traditional medicine for their primary health care. This is attributed to the high level of poverty in the district, long distances travelled to access free health services and prolonged drug stockouts [21, 22].

### Sample size and sampling procedures

A sample size of 245 respondents was calculated using the formula suggested by Krejcie and Morgan [23]. Due to COVID-19 restrictions and limited resources, we interviewed only 151 respondents (21 traditional medicine practitioners and 130 common people, i.e. local people who regularly use plants for medicinal purposes). These respondents were both females and male aged 18 years and above.

### Study design, selection of study sites and participants

Field survey for this study was conducted from February 2020 to September 2020 using a cross-sectional study design. Three sub-counties of Tororo district (Fig. 1) namely Eastern division, Kirewa, and Paya were randomly selected. Two parishes were randomly selected from each division and eventually, two villages were considered. This gave a total of 12 villages. In each village, herbalists were purposively sampled based on their reputation in the community to treat symptoms of malaria. As the key informants, herbalists were identified using snow balling method based on the principle of saturation [24]. Using this method, once an herbalist was identified and interviewed, they were asked to refer the research team to another herbalist within their networks. The subsequent herbalist then referred us to the next herbalist in their networks until saturation was reached. From each village, 10–15 respondents were interviewed altogether. Experienced non-herbalists were randomly selected to participate in the study after obtaining their prior informed consent.

### Ethnobotanical data collection

A pilot study was undertaken in February 2020 to introduce the study to the local area administration, seek their permission to conduct the study and pre-test the study tool. Data were collected from the respondents following guidelines of conducting research during the COVID-19 pandemic established by the Uganda National Council of Science and Technology [25]. Data were collected using a semi-structured questionnaire which was translated into Japadhola, the principal language spoken in Tororo district. The questionnaire included questions on the respondent’s biodata, knowledge on signs and symptoms of malaria, harvesting, preparation, administration and dosage of malaria herbal medicines (Additional file 1: S1). Questions on the existing knowledge, attitudes and practices related to malaria recognition, control and treatment in Tororo district were also included. Three focus group discussions were held with community members (one per sub-county) to complement the questionnaire survey. Plants mentioned by respondents were identified during guided field walks with the informants [26]. Voucher specimen of each plant species were prepared for correct botanical identification and deposited at the Makerere University Herbarium. Species nomenclature follows the flora for tropical East Africa and was verified using the Plants of the World Online (POWO) database (https://powo.science.kew.org).

### Data analysis

Numerical data were entered into Microsoft Excel spread sheet, coded, and exported to SPSS software (version 26, SPSS Inc.) for analysis. Descriptive statistics such as percentages and frequencies were used to summarize ethnobotanical and respondents’ socio-demographic data. Further, ethnobotanical data were used to calculate informant consensus factor as well as perform paired comparison and preference ranking.

#### Informant consensus factor

To determine the homogeneity of the ethnobotanical information collected from the respondents, the Informant Consensus Factor (ICF) was computed using formula 1 [27]:

1 $$\mathrm{ICF}=\frac{\mathrm{Nur}-\mathrm{Nt}}{\mathrm{Nur}-1},$$

where “$\mathrm{Nur}$” refers to the total number of use reports for each disease cluster and “$\mathrm{Nt}$” refers the total number of species in each use category. The ICF values range from 0 to 1. High ICF values (close to 1) are obtained when only a few plant species are reported to be used by a high proportion of informants to treat a particular disease and this implies that there is a well-defined mechanism in the community of sharing information between informants. Low ICF values (close to 0) are obtained when many plant species are reported to be used by a high proportion of informants to treat a particular disease and this implies that there is no well-defined mechanism in the community of sharing information between informants.

#### Preference ranking

Preference ranking was performed as reported by Martin [28]. When a variety of plant species are utilized to treat the same health problem, individuals prefer one over the other. Key informants were given the task of comparing the given medicinal plants based on their values, with the highest number (5) given to medicinal plants which they preferred to be the most effective in treating malaria and the lowest number (1) given to those plants that they preferred to be the least effective in treating malaria [29].

#### Paired comparison of medicinal plants

A paired comparison was made for five medicinal plants used to treat malaria in the study area. Ten reputable herbalists were requested to rank the species based on their efficiency in management of malaria as follows: 1 = least, 2 = good, 3 = very good and 4 = excellent [29].

## Results

### Sociodemographic characteristics

The respondents were distributed by gender with 41.1% females and 58.9% males. The majority of these (94%) were married. The major occupation was subsistence farming (61.6%), followed by casual labour for wages (22.5%). The respondents had a median age of 46.0 years, and a significant percentage (65%) had attained only primary education (Table 1).

**Table 1:** Socio-demographic characteristics of respondents from Tororo district

Characteristics	Frequency	Percentage (%)
Sex		
Female	62	41.1
Male	89	58.9
Age group		
18–34	65	43.0
35–59	75	49.7
60+	11	7.3
Marital status		
Not married	9	6.0
Married	142	94.0
Religion		
Catholic	45	29.6
Anglican	44	29.0
Moslem	32	21.5
Pentecostal	25	16.7
None	5	3.2
Ethnicity		
Japadhola	101	67.0
Itesot	32	21.0
Other	18	12.0
Highest level of education		
No formal education	3	2.0
Primary	98	65.0
Secondary	47	31.0
Tertiary university	3	2.0
Occupation		
Casual workers for wages	34	22.5
Formal employment/professional	12	7.9
Subsistence farmer	93	61.6
Unemployed	12	7.9
Source of traditional knowledge		
Parents and relatives	105	69.5
Other community members	44	29.1
Traditional medicine association	2	1.4

### Knowledge on malaria, its symptoms and treatment-seeking behaviour of patients

**Fig. 2:**
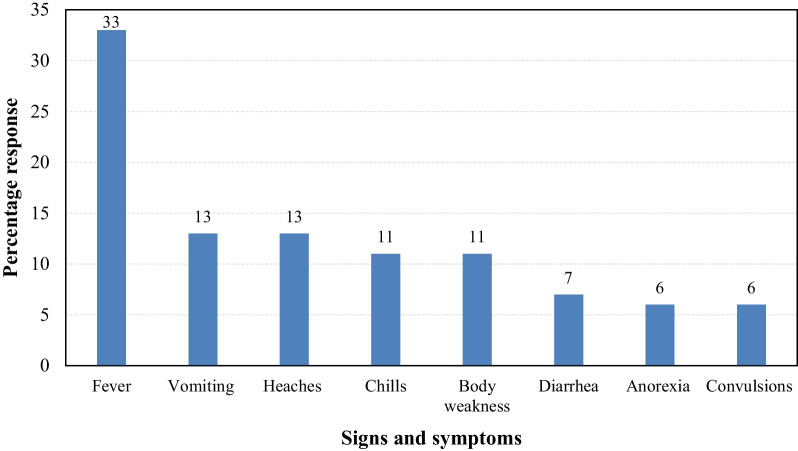
Reported signs and symptoms of malaria by respondents in Tororo district, Eastern Uganda

Malaria appeared to be prevalent and well understood by the respondents in Tororo district. Most respondents (90%) had suffered from malaria in the last six months before the date of the interview. The respondents also mentioned the correct signs and symptoms of malaria (Fig. 2). Fever (33%) was the main sign of malaria reported by the respondents, followed by vomiting (13%) and body weakness (11%). Other signs and symptoms of malaria reported were headache (13%), diarrhoea (7%), convulsions (6%), loss of appetite (6%) and body chills (11%).

**Fig. 3:**
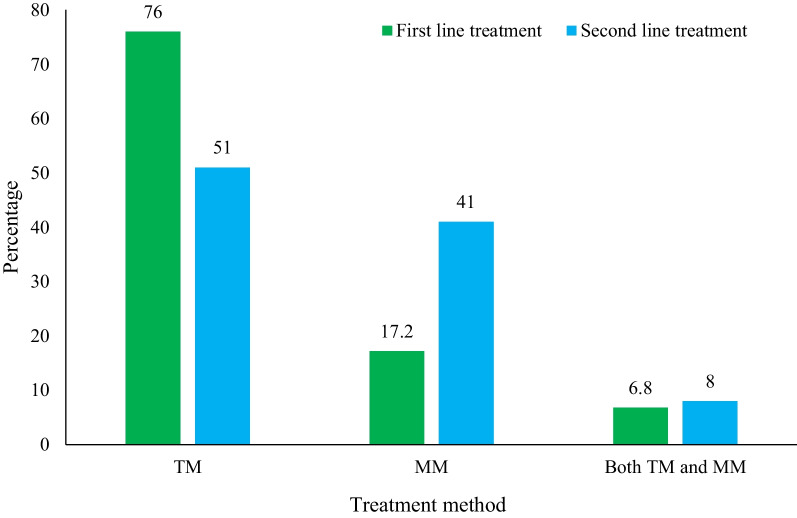
Treatment options used by malaria patients in the studied communities of Tororo district, Eastern Uganda. TM: traditional medicine; MM: modern medicine

On developing malaria, most respondents (76%) reported that they used traditional medicine (TM) alone as the first line of treatment compared to 17.2% who used modern medicine (MM) alone. When they failed to improve, they switched to MM. Thus, on re-treatment, the number of people that used TM alone decreased to 51%, while 41% used MM (Fig. 3). In clinical practice, a first-line treatment/first-line therapy is the treatment that is accepted as best for the initial treatment of a condition or disease. In the context of our study, malaria patients tend to use herbal remedies (TM) as the first treatment for malaria before attempting to use MM. In case it fails, the second treatment sought after is MM.

### Plant species used in preparation of herbal remedies for malaria treatment in Tororo district

**Fig. 4:**
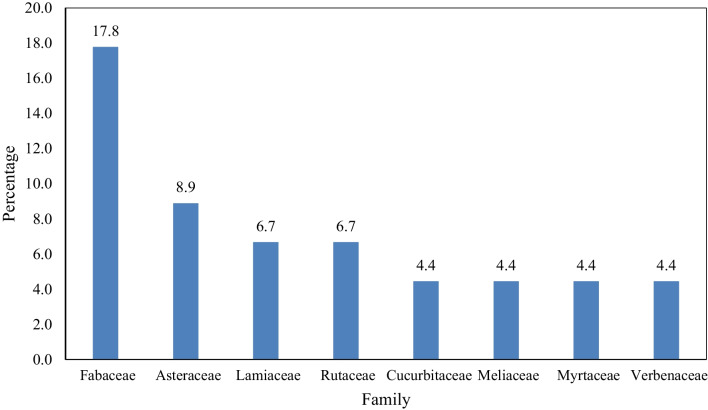
Distribution of medicinal plant species for management of malaria in Tororo district by families

Forty-five plant species were mentioned by respondents in this study to be used in preparation of herbal remedies for management of symptoms of malaria (Table 2). Of the inventoried species, nine were mentioned by six or more people. These are; *Vernonia amygdalina* Delile (58), *Chamaecrista nigricans* (Vahl.) Greene (14), *Aloe nobilis* (L.) Burman. (13), *Warburgia ugandensis* Sprague (12), *Abrus precatorius* L. (11), *Kedrostis foetidissima* Cogn. (10), *Senna occidentalis* L., *Azadirachta indica* (7 each), and *Mangifera indica* L. (6). The species were distributed as trees (37.7%), shrubs (26.7%) and herbs (35.6%) by growth habit. These species were from 26 families and 44 genera. Fabaceae (17.8%), Asteraceae (8.9%), Lamiaceae and Rutaceae (6.7% each) were the most represented families (Fig. 4). The ICF for malaria calculated was 0.76, implying that there is considerable agreement among the community members in the medicinal plants used in management of malaria. For preference ranking, *Vernonia amygdalina* Delile, *Chamaecrista nigricans* (Vahl.) Greene and *Aloe nobilis* (L.) Burman. were ranked first, second and third, respectively (Table 3). The results of paired comparison test for the five frequently mentioned plant species, respondents (10) selected *Vernonia amygdalina* Delile first, followed by *Chamaecrista nigricans* (Vahl.) Greene, *Warburgia ugandensis* Sprague, *Aloe nobilis* (L.) Burman. and *Abrus precatorius* L. (Table 4).

**Table 2:** Medicinal plants used in Eastern Division, Kirewa, and Paya sub-counties of Tororo district, Uganda, for treating malaria (*n* = 45)

S/N	Name	Local name	Family	Voucher no.	Habit	Part(s) used	Administration method	Frequency
1	*Vernonia amygdalina* Delile	Muluswa	Asteraceae	AN01	Shrub	Leaves	Oral	58
2	*Chamaecrista nigricans* (Vahl.) Greene	Achwa	Fabaceae	AN02	Herb	Leaves	Oral	14
3	*Aloe nobilis* (L.) Burman	Atworo	Asphodelaceae	AN03	Herb	Leaves	Oral	13
4	*Warburgia ugandensis* Sprague	Atiko	Canellaceae	AN04	Tree	Leaves	Oral	12
5	*Abrus precatorius* L	Osito	Fabaceae	AN05	Herb	Leaves	Oral	11
6	*Kedrostis foetidissima* Cogn	Nyamikesi	Cucurbitaceae	AN06	Herb	Root bark	Oral	10
7	*Senna occidentalis* L	Yeke yeke	Fabaceae	AN07	Shrub	Leaves	Oral	7
8	*Azadirachta indica* A. Juss	Arubaine	Meliaceae	AN08	Tree	Leaves	Oral	7
9	*Mangifera indica* L	Mayembe	Anacardiaceae	AN09	Tree	Leaves	Oral	6
10	*Clerodendrum myricoides* (Hochst.) Vatke	Okwero	Verbenaceae	AN10	Shrub	Stem, fruits, roots, leaves	Oral	3
11	*Carissa spinarum* L	Ochwoga	Apocynaceae	AN11	Shrub	Roots	Oral	3
12	*Bidens pilosa* L	Sere	Asteraceae	AN12	Herb	Leaves	Topical bath	3
13	*Citrus limon* (L.) Burm	Nimoo	Rutaceae	AN13	Tree	Leaves, roots	Steam bath	3
14	*Eucalyptus camaldulensis* Denhn	Kalitusi	Myrtaceae	AN14	Tree	Leaves	Topical bath	3
15	*Tamarindus indica* L	Chwa	Fabaceae	AN15	Tree	Leaves, stem	Oral	2
16	*Psidium guajava* L	Mapeera	Myrtaceae	AN16	Shrub	Leaves	Oral	2
17	*Persea americana* Mill	Avocado	Lauraceae	AN17	Tree	Leaves	Oral	2
18	*Momordica foetida* K. Schum	Woyo	Cucurbitaceae	AN18	Herb	Leaves	Oral	1
19	*Clematis hirsuta* Perr. & Guill	Adwe	Ranunculaceae	AN19	Herb	Leaves	Oral	1
20	*Fagaropsis angolensis* (Engl.) Dale	Rokoo	Rutaceae	AN20	Tree	Roots, leaves, fruits, seeds	Oral	1
21	*Microglossa densiflora* Hook.f	Omeryidiegi	Asteraceae	AN21	Herb	Root bark	Oral	1
22	*Solanum ptychanthum* Dunal	Ochoki	Solanaceae	AN22	Shrub	Fruits	Oral	1
23	*Leonotis nepetifolia* (L.) R.Br	Odhudho/Othutho	Lamiaceae	AN23	Herb	Leaves	Oral	1
24	*Cannabis sativa* L	Misaala	Cannabaceae	AN24	Herb	Leaves	Topical bath	1
25	*Cissampelos mucronata* A. Rich	Masu	Menispermaceae	AN25	Herb	Roots, leaves	Oral	1
26	*Tetradenia riparia* (Hochst.) Codd	Aboke	Lamiaceae	AN26	Shrub	Leaves	Oral	1
27	*Oldenlandia herbacea* (L.) Roxb	Alwari	Rubiaceae	AN27	Herb	Roots	Oral	1
28	*Melia azedarach* L	Lira	Meliaceae	AN28	Tree	Leaves, roots	Oral	1
29	*Albizia coriaria* Welw. ex Oliv	Oberi	Fabaceae	AN29	Tree	Stem	Oral	1
30	*Ocimum basilicum* L	Yathi ajwoka	Lamiaceae	AN30	Herb	Leaves	Topical bath	1
31	*Toddalia asiatica* (L.) Lam	Thwolikiluwi	Rutaceae	AN31	Shrub	Leaves	Oral	1
32	*Harrisonia abyssinica* Oliv	Pedo	Simaroubaceae	AN32	Shrub	Fruits	Oral	1
33	*Acacia campylacantha* Hochst.ex A. Rich	Mugogwe	Fabaceae	AN33	Tree	Root bark	Oral	1
34	*Urena lobata* L	Mbirambira	Malvaceae	AN34	Shrub	Leaves	Oral	1
35	*Annona senegalensis* Pers	Obolo	Annonaceae	AN35	Tree	Root wood	Oral	1
36	*Vitex doniana Sweet*	Yuelo/Uwelo	Verbenaceae	AN36	Tree	Leaves, roots	Oral	1
37	*Ficus cyathistipula* Warb	Bongi	Moraceae	AN37	Tree	Leaves	Oral	1
38	*Carica papaya* L	Mapapali	Caricaceae	AN38	Tree	Leaves	Oral	1
39	*Vigna unguiculata* (L.) Walp	Boo	Fabaceae	AN39	Herb	Leaves	Oral	1
40	*Panicum maximum* Jacq	Thiwi odunyo	Poaceae	AN40	Herb	Leaves	Oral	1
41	*Moringa oleifera* Lam	Moringa	Moringaceae	AN41	Tree	Leaves, roots	Topical bath	1
42	*Desmodium velutinum* (Willd.) DC	Sirangende	Fabaceae	AN42	Herb	Root bark	Oral	1
43	*Punica granatum* L	Nkomamawanga	Lythraceae	AN43	Shrub	Leaves, roots	Oral	1
44	*Vernonia adoensis* Sch. Bip. ex Walp	Muluswa matari	Asteraceae	AN44	Shrub	Leaves	Oral	1
45	*Grevillea robusta* A. Cunn. ex R.Br	Grevillia	Proteaceae	AN45	Tree	Leaves	Oral	1

**Table 3:** Preference ranking of medicinal plants used for treating malaria in Tororo District, Eastern Uganda

Name of species	Respondents (R_1_–R_7_)							Score	Rank
	R_1_	R_2_	R_3_	R_4_	R_5_	R_6_	R_7_		
*Abrus precatorius* L	2	3	3	2	3	3	3	19	5th
*Aloe nobilis* (L.) Burman	4	3	4	4	4	2	4	25	3rd
*Chamaecrista nigricans* (Vahl.)	2	5	5	3	5	2	5	27	2nd
*Kedrostis foetidissima* Cogn	4	2	2	3	2	3	2	17	6th
*Vernonia amygdalina* Delile	5	3	4	5	5	4	5	31	1st
*Warburgia ugandensis* Sprague	3	2	3	4	3	4	3	22	4th

**Table 4:** Paired comparison on five commonly used medicinal plants used for treating malaria in Tororo District, Eastern Uganda

Name of species	Respondents										Score	Rank
	R_1_	R_2_	R_3_	R_4_	R_5_	R_6_	R_7_	R_8_	R_9_	R_10_		
*Abrus precatorius* L	2	2	3	3	2	2	2	2	3	2	23	5th
*Aloe nobilis* (L.) Burman	4	3	2	2	3	3	3	3	3	3	29	3rd
*Chamaecrista nigricans* (Vahl.)	2	3	3	3	3	3	4	3	4	4	32	2nd
*Vernonia amygdalina* Delile	4	4	3	4	4	4	4	4	4	3	38	1st
*Warburgia ugandensis* Sprague	3	3	4	3	3	2	3	2	3	3	29	3rd

### Preparation and administration of herbal medicine for management of malaria

**Fig. 5:**
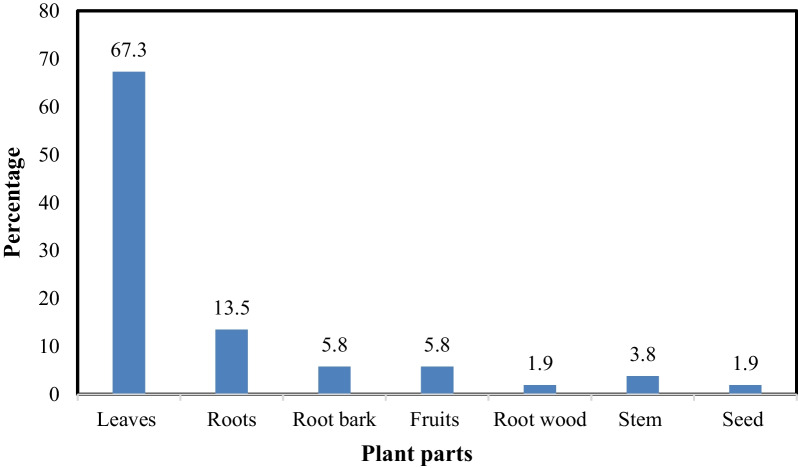
Plant parts used in the treatment of malaria in Tororo district, Uganda

Leaves (67.3%) were the most commonly used plant part, followed by roots (13.5%), root bark (5.8%) and fruits (5.8%) (Fig. 5). The herbal remedies are prepared through maceration (56%) and as decoctions (34%). However, they can also be powdered (6%) or prepared as infusions (4%).

The herbal medicines were majorly administered orally (86.7%). Other routes of administration were topical baths (11.1%) and steam baths (2.2%). The medicaments were mostly processed and used when needed, and were rarely preserved. The most used packaging materials for liquid forms were plastic bottles (0.5–1L) and small jerricans (1–3L), whereas solids and powders were packed in polyethene bags. The plant materials were collected from the wild (46%), gardens (30%), compounds (14%) and other places (11%) such as roadsides and swamps. Most respondents (78.8%) reported that they grow the plants while others (10%) said that they purchase the herbs.

## Discussion

Malaria is still a disease of public health importance in Tororo district. Our results indicate that people are familiar with malaria, and can correctly recognize it basing on the signs and symptoms. Majority of the people used TM (as opposed to MM) to treat malaria. A study on treatment-seeking behaviour and practices among caregivers of children aged 5 years with presumed malaria in rural Namutumba district (a nearby district in Eastern Uganda) showed that only 36.1% of the patients took herbal medicines. Most of them sought MM with nearly all the patients who used TM also taking modern antimalarials [30]. Further, 79.2% of the patients who used herbal medicines to treat malaria also received artemether–lumefantrine in in the same study area [30]. Hasabo et al. [31] in their study in South Sudan reported that when people fell sick from malaria, 78% of the patients sought treatment from the nearby primary health centre (MM). Although we could not ascertain the real drivers of the high use of TM in this study area, we think that apart from poverty that makes the conventional drugs unaffordable [32], the high travel restrictions instituted during the COVID-19 pandemic especially on border districts like Tororo could have forced patients to find alternative therapies of which herbal medicines are the most readily available and affordable. Additionally, they were also perceived to be efficacious and safe. The form of treatment chosen for malaria depended on its perceived severity. For the most part, uncomplicated malaria is often treated using a mixture of traditional and modern methods, and this is a common practice throughout Africa [33, 34].

This study identified 45 plant species used in Tororo district, Eastern Uganda for managing malaria. Most of the species identified has been cited for treatment of malaria in other parts of Uganda as well as other countries. For example, *Albizia coriaria*, *Momordica foetida* and *Carica papaya* are used elsewhere in Uganda [18, 30, 35, 36], Cameroon [37] and Zimbabwe [38]. *Harrisonia abyssinica* is used in Tanzania [39] and South Africa [40], while *Tamarindus indica*, *Carica papaya* and *Ocimum basilicum* are used in Indonesia [41]. With the exception of a few species such as *Kedrostis foetidissima*, *Mangifera indica* and *Carissa spinarum*, most of the plants indicated by the respondents in Tororo are used in the management of malaria and its symptoms in the neighbouring Kenya [5]. The high ICF (0.76) implies that there is sharing of indigenous knowledge related to medicinal plants use in malaria management among the community members. Hence, it is likely that the same plant species are used in the preparation of herbal medicines for malaria by majority of the people in Tororo. The dominant source of collection of medicinal plants from the wild highlights the dependence of the traditional healers on wild crafted materials. However, they are also interested in conserving the species as some collect them from gardens and the compound. The species mentioned by most people could be considered to be efficacious for the treatment of malaria and were prioritized on this basis for further analysis. A review of the available literature revealed that of the 45 plant species used in the traditional treatment of malaria in Tororo, 17 species have been evaluated for the antimalarial/antiplasmodial activity using different assays. These species possess acceptable preclinical safety and efficacy (Table 5). This confirms that indeed the reported medicinal plants possess antimalarial properties which can be further investigated for development of new antimalarial drugs.

**Table 5:** Literature on antiplasmodial/antimalarial activities and toxicity of extracts and isolated compounds of the plants identified in Tororo District, Eastern Uganda

Plant name	Part used	Solvent used	Antiplasmodial (IC_50_ μg/ml)/antimalarial (*Plasmodium* strain) activities	Active phytochemicals	Toxicity	References
*Abrus precatorius* L	Leaves	Methanol	85.59 (D6), > 100 (W2)	Abruquinone B isolated from the aerial parts; showed antiplasmodial activity (IC_50_ = 1.5 μg/ml)	Two main cytotoxic constituents of leaf extract were Stigmasterol hemihydrate and β-monolinolein (IC_50_ = 74.2 and 13.2 µg/ml), respectively, in MDA-MB-231 breast cancer cells and cytotoxic activities. Abruquinone B was cytotoxic towards KB & BC cell lines (IC_50_:13.0 ± 19.8 μg/ml)	[42–44]
*Albizia coriaria* Welw. ex Oliver	Stem bark	Methanol	15.2 (D6); 16.8 (W2)	Triterpenoids, lupeol, lupenone	Cytotoxic to the human glioblastoma cell line U87 CD4 CXCR4 (CC_50_ = 6.4 and 14.9 μg/ml for ethanol and DMSO extracts	[45, 46]
*Azadirachta indica* A. Juss	Leaves	Water, methanol	17.9 (D6); 43.7 (W2)	Terpenoids, isoprenoids, gedunin, limonoids: khayanthone, meldenin, nimbinin	Cytotoxicity LD_50_ of 101.26 and 61.43 µg/ml for water and methanol extracts	[47–51]
*Bidens pilosa* L	Leaves	Dichloromethane, water, methanol	8.5, 5, 11, 70 (D10)	No reports	Hydro and ethanol extracts are not toxic in mice (LD_50_ = 12.3 and 6.2 g/kg bw), respectively. Safe in humans	[52–54]
*Carica papaya* L	Leaves	Ethyl acetate	2.96 (D10), 3.98 (DD2), 0.2 uM (carpaine)	Carpaine	Carpaine has high selectivity (106) and is nontoxic to normal red blood cells and rat skeletal myoblast (L6) cells	[55–57]
*Cissampelos mucronata* A. Rich	Root bark, root	Methanol, ethyl acetate	8.8 (D6); 9.2 (W2). Root extract- < 3.91 (D6), 0.24 (W2) for curine	Benzylisoquinoline alkaloids, curine	Slightly to moderately toxic LD_50_ = 288.53 mg/kg for the ethanol root extract and 8500 mg/kg for the methanol leaf extract	[45, 58–62]
*Clerodendrum myricoides*	Root bark	Ethanol chloroform	4.7 (D6); 8.3 (W2) > 10 (D6)	No reports	Cytotoxicity = IC_50_ > 20.0 μg/ml	[63, 64]
*Harrisonia abyssinica* Olive	Roots	Water, methanol	4.4 (D6), 10.25 (W2); 89.74, 79.50 (ENT 30); 86.56	Limonoids, steroids	Slightly to moderately toxic with LD_50_ of 234.71and 217.34 µg/ml for water and methanol extracts in mice	[50, 51, 60, 62]
*Melia azedarach* L	Leaves	Methanol, DCM	55.1 (3D7), 19.1 (W2); 28	No reports	No reports of leaf toxicity	[65, 66]
*Momordica foetida* Schumach	Shoot	Water	6.16 (NF54); 0.35 (FCR3)	Saponins, alkaloids, cardiac glycosides	No pronounced toxicity against human hepatocellular (HepG2) and human urinary bladder carcinoma (ECV-304, derivative of T-24) cells	[67–69]
*Ocimum basilicum* L	Leaves, whole plant	Ethanol	68.14 (3D7); 67.27 (INDO)	No reports	LD_50_ in rats was greater than 5000 mg/kg body weight. Not toxic, generally safe, LD_50_ = 100–5000 mg/kg body weight	[60, 62, 64, 70, 71]
*Senna occidentalis* L	Leaves	Dimethyl sulfoxide, ethanol	48.80 (3D7), 54.28 (NIDO); < 3	Quinones	Slightly to moderately toxic LD_50_ of leaf and stem extracts = 5 g/kg in mice	[60, 62, 64, 72–74]
*Solanum incanum* L	Leaves	Chloroform/methanol	31% parasite suppression	No report	No mortality and overt toxicity in mice at the limit dose of 2 g/kg: LD_50_ of both leaf and root hydromethanol extracts > 2 g/kg in mice	[75]
*Tamarindus indica* L	Stem bark	Water	25.1% parasite suppression at 10 mg/kg (*P. berghei*)	Saponins (leaves), tannins (fruits)	Not toxic	[76, 77]
*Toddalia asiatica (*L.) Lam	Root bark, fruits and leaves	Methanol, water, ethyl acetate, hexane	6.8 (D6); 13.9 (W2). Ethyl acetate fruit extract (1.80 mg/ml), root bark aqueous (2.43) (W2)	Furoquinolones (nitidine, 5,6-dihydronitidine), coumarins	Slightly to moderately toxic Acute and cytotoxicity of the extracts, with the exception of hexane extract from the roots showed LD_50_ > 1000 mg/kg and CC_50_ > 100 mg/ml, respectively	[45, 60, 62, 78]
*Vernonia amygdalina* Del	Leaves	Methanol/dichloromethane, ethanol	2.7 (K1), 9.83. In vivo parasite suppression of between 57.2–72.7% in combination with chloroquine	Vernolepin, vernolin, vernolide, vernodalin and hydroxy vernodalin, steroid glucosides	Petroleum ether extract shows strong cytotoxicity	[69, 79–83]
*Warburgia ugandensis* Sprague	Stem bark	Methanol, water, dichloromethane	6.4 (D6); 6.9 (W2), 12.9 (D6); 15.6 (W2) 69% parasite suppression	Coloratane sesquiterpenes, e.g., muzigadiolide	Cytotoxic to the human glioblastoma cell line U87 CD4 CXCR4 (CC_50_ = 7.2 and 2.0 μg/ml for ethanol and DMSO extracts	[45, 46, 79, 84–86]

*Plasmodium falciparum* isolates: Chloroquine sensitive strains are D6, 3D7, D10, FCR3, and NF54; Chloroquine resistant are Dd2, ENT30, FCR3, K1, NIDO, V1/S and W2

The oral route was the most used mode of drug administration. This could partly be attributed to the fact that oral dosage forms are easy to prepare and administer [5]. Like in other communities, appropriate dose determination was a challenge as many herbalists just gave estimates using cups and spoons. The preparation and packaging procedures were also prone to contamination and there was no evidence of consistency in the preparation procedures used. Hence, there is a need to sensitize the respondents about standardization procedures and good manufacturing practices so as to enhance the quality of their traditional medicine.

## Conclusion

This study identified 45 medicinal plants majorly from family Fabaceae and Asteraceae used in preparation of traditional medicines for management of symptoms of malaria in Tororo district. The phytochemical constituents, antiplasmodial and antimalarial activity as well as toxicity profiles of the unstudied species with high percentage use values should be investigated to validate their uses in the management of malaria in an effort to discover novel antimalarial drugs.

## Supplementary Information

**Additional file 1.** **S1.** Questionnaire used in the ethnobotanical survey of medicinal plants used for treatment of malaria by indigenous communities of Tororo District, Eastern Uganda.

## Data Availability

The raw data supporting the conclusions of this study are available upon request from the corresponding author.

## Abbreviations

ICF: Informant consensus factor
MM: Modern medicine
TM: Traditional medicine

## References

[1] WHO. World Malaria Report 2021. World Health Organization 2021. https://www.who.int/teams/global-malaria-programme/reports/world-malaria-report-2021. Accessed 20 May 2022.

[2] Alonso S, Chaccour CJ, Elobolobo E, Nacima A, Candrinho B, Saifodine A, Saute F, Robertson M, Zulliger R (2019). The economic burden of malaria on households and the health system in a high transmission district of Mozambique. Malar J.

[3] Kayiba NK, Yobi DM, Devleesschauwer B, Mvumbi DM, Kabututu PZ, Likwela JL, Kalindula LA, DeMol P, Hayette MP, Mvumbi GL, Lusamba PD, Beutels P, Rosas-Aguirre A, Speybroeck N (2021). Care-seeking behaviour and socio-economic burden associated with uncomplicated malaria in the Democratic Republic of Congo. Malar J.

[4] Murphy MW, Dunton RF, Perich MJ, Rowley WA (2001). Attraction of *Anopheles* (Diptera: culicidae) to volatile chemicals in Western Kenya. J Med Entomol.

[5] Omara T (2020). Antimalarial plants used across Kenyan communities. Evid Based Complement Alternat Med.

[6] WHO. Global Malaria Programme. Artemisinin resistance and artemisinin-based combination therapy efficacy. 2018. https://www.who.int/docs/default-source/documents/publications/gmp/who-cds-gmp-2018-26-eng.pdf#:~:text=WHO%20recommends%20artemisinin-based%20combination%20therapies%20%28ACTs%29%20for%20the,treatment%20of%20malaria%20is%20a%20global%20health%20priority. Accessed 28 May 2022.

[7] Daher A, Aljayyoussi G, Pereira D (2019). Pharmacokinetics/pharmacodynamics of chloroquine and artemisinin-based combination therapy with primaquine. Malar J.

[8] Ataba E, Dorkenoo AM, Nguepou CT, Bakai T, Tchadjobo T, Kadzahlo KD, Yakpa K, Atcha-Oubou T (2022). Potential emergence of *Plasmodium* resistance to artemisinin induced by the use of *Artemisia annua* for malaria and COVID-19 prevention in Sub-African Region. Acta Parasitol.

[9] Uganda Ministry of Health, National Malaria Control Division, Surveillance Monitoring & Evaluation Unit. National Malaria Annual Report 2017–2018, Kampala, Uganda. 2019. https://www.health.go.ug/sites/default/files/Malaria%20Annual%20Report%20July%202017%20web%20%282%29_0.pdf. Accessed 28 May 2022.

[10] CDC. Treatment of Malaria: Guidelines for Clinicians (United States). 2020. https://www.cdc.gov/malaria/resources/pdf/Malaria_Treatment_Guidelines.pdf. Accessed 28 May 2022.

[11] Lu G, Cao Y, Chen Q, Zhu G, Müller O, Cao J (2022). Care-seeking delay of imported malaria to China: implications for improving post-travel healthcare for migrant workers. J Travel Med.

[12] Jagannathan P, Kakuru A (2022). Malaria in 2022: increasing challenges, cautious optimism. Nat Commun.

[13] Hodoameda P, Duah-Quashie NO, Quashie NB (2022). Assessing the roles of molecular markers of antimalarial drug resistance and the host pharmacogenetics in drug-resistant malaria. J Trop Med.

[14] Mawanda P. Malaria Cases On the Rise- Ministry of Health. 2022. https://ugandaradionetwork.com/story/malaria-cases-on-the-rise-ministry-of-health. Accessed 28 May 2022.

[15] Malaria Consortium. Coverage and quality of seasonal malaria chemoprevention supported by Malaria Consortium in 2021: Results from Burkina Faso, Chad, Mozambique, Nigeria, Togo, and Uganda. 2021. https://www.malariaconsortium.org/media-downloads/1582/Coverage%20and%20quality%20of%20seasonal%20malaria%20chemoprevention%20supported%20by%20Malaria%20Consortium%20in%202021:%20Results%20from%20Burkina%20Faso,%20Chad,%20Mozambique,%20Nigeria,%20Togo,%20and%20Uganda. Accessed 28 May 2022.

[16] Khanal P (2021). Antimalarial and anticancer properties of artesunate and other artemisinins: current development. Monatsh Chem.

[17] Muangphrom P, Seki H, Fukushima EO, Muranaka T (2016). Artemisinin-based antimalarial research: application of biotechnology to the production of artemisinin, its mode of action, and the mechanism of resistance of *Plasmodium* parasites. J Nat Med.

[18] Okello D, Kang Y (2019). Exploring antimalarial herbal plants across communities in Uganda based on electronic data. Evid Based Complement Alternat Med.

[19] Oguttu DW, Matovu JKB, Okumu DC, Ario AR, Okullo AE, Opigo J, Nankabirwa V (2017). Rapid reduction of malaria following introduction of vector control interventions in Tororo District, Uganda: a descriptive study. Malar J.

[20] Musiime AK, Smith DL, Kilama M, Rek J, Arinaitwe E, Nankabirwa JI (2019). Impact of vector control interventions on malaria transmission intensity, outdoor vector biting rates and *Anopheles* mosquito species composition in Tororo, Uganda. Malar J.

[21] The Independent. Tororo leaders meet to find solutions for rising poverty, poor roads, low rank hospital. 2021. https://www.independent.co.ug/tororo-leaders-meet-to-find-solutions-for-rising-poverty-poor-roads-low-rank-hospital/. Accessed 30 Apr 2023.

[22] Cyrus D. Integration of the Private Health Providers into the district health system: an action research in Tororo district, Uganda. 2019. http://speed.musph.ac.ug/wp-content/uploads/2020/07/Action-Research-in-Tororo-District-David.pdf. Accessed 30 Apr 2023.

[23] Krejcie RV, Morgan DW (1970). Determining sample size for research activities. Educ Psychol Measur.

[24] Johnson TP. Snowball sampling: introduction. Wiley StatsRef: Statistics Reference Online. In: Encyclopedia of Biostatistics. 2014. 10.1002/9781118445112.stat05720.

[25] UNCST. Guidelines of conducting research during the COVID-19 pandemic. https://www.unhro.org.ug/assets/images/resources/covidnationalguidelines.pdf. 2020.

[26] Dossou AJ, Fandohan AB, Omara T, Gbenou J (2022). Traditional knowledge and phytochemical screening of plants used in snakebite prevention in Benin. Bull Natl Res Cent.

[27] Trotter RJ, Logan MH, Etkin NL (1986). Informant consensus. A new approach for identifying potentially effective medicinal plants. Plants in indigenous medicine and diet.

[28] Martin G (1995). Ethnobotany: a methods manual.

[29] Yimam M, Yimer SM, Beressa TB (2022). Ethnobotanical study of medicinal plants used in Artuma Fursi district, Amhara Regional State. Ethiopia Trop Med Health.

[30] Lee YJ, Adusumilli G, Kazungu R, Anywar G, Kyakulaga F, Katuura E, Parikh S, Willcox M (2019). Treatment-seeking behavior and practices among caregivers of children aged ≤5 y with presumed malaria in rural Uganda. Trans R Soc Trop Med Hyg.

[31] Hasabo EA, Khalid RI, Mustafa GE, Taha RE, Abdalla RS, Mohammed RA, Haroun MS, Adil R, Khalil RA, Mansour RM, Mohamed RK, Awadalla H (2022). Treatment-seeking behaviour, awareness and preventive practice toward malaria in Abu Ushar, Gezira state, Sudan: a household survey experience from a rural area. Malar J.

[32] Obakiro SB, Kiyimba K, Napyo A, Kanyike AM, Mayoka WJ, Nnassozi AG, Aguti B, Akech GM, Waako JP (2021). Appropriateness and affordability of prescriptions to diabetic patients attending a tertiary hospital in Eastern Uganda: a retrospective cross-sectional study. PLoS ONE.

[33] Beiersmann C, Sanou A, Wladarsch E, De Allegri M, Kouyaté B, Müller O (2007). Malaria in rural Burkina Faso: local illness concepts, patterns of traditional treatment and influence on health-seeking behaviour. Malar J.

[34] Diallo D, Graz B, Falquet J, Traoré AK, Giani S, Mounkoro PP, Berthé A, Sacko M, Diakité C (2006). Malaria treatment in remote areas of Mali: use of modern and traditional medicines, patient outcome. Trans R Soc Trop Med Hyg.

[35] Adia MM, Anywar G, Byamukama R, Kamatenesi-Mugisha M, Sekagya Y, Kakudidi EK, Kiremire BT (2014). Medicinal plants used in malaria treatment by Prometra herbalists in Uganda. J Ethnopharmacol.

[36] Anywar G, Byamukama R, vant Klooster CIEA, Wilcox M, Nalumansi P, de Jong J, Rwaburindori P, Kiremire BT (2016). Medicinal plants used in the treatment and prevention of malaria in Cegere sub-county, Northern Uganda. J Ethnobot Appl Res..

[37] Pierre S, Toua V, Tchobsala, Tchuenguem FF, Alexandre-Michel NN, Jean M (2011). Medicinal plants used in traditional treatment of malaria in Cameroon. J Ecol Nat Environ..

[38] Ngarivhume T, Van't Klooster CI, de Jong JT, Van der Westhuizen JH (2015). Medicinal plants used by traditional healers for the treatment of malaria in the Chipinge district in Zimbabwe. J Ethnopharmacol.

[39] Chhabra SC, Mahunnah RL, Mshiu EN (1993). Plants used in traditional medicine in eastern Tanzania. VI. Angiosperms (Sapotaceae to Zingiberaceae). J Ethnopharmacol.

[40] Watt JM, Breyer-Brandwijk MG (1962). The medicinal and poisonous plants of Southern and Eastern Africa.

[41] Taek MM, Bambang PEW, Mangestuti A (2018). Plants used in traditional medicine for treatment of malaria by Tetun ethnic people in West Timor Indonesia. Asian Pac J Trop Med.

[42] Gathirwa JW, Rukunga GM, Mwitari PG, Mwikwabe NM, Kimani CW, Muthaura CN, Kiboi DM, Nyangacha RM, Omar SA (2011). Traditional herbal antimalarial therapy in Kilifi district. Kenya J Ethnopharmacol.

[43] Sofi MS, Sateesh MK, Bashir M, Ganie MA, Nabi S (2018). Chemopreventive and anti-breast cancer activity of compounds isolated from leaves of *Abrus precatorius* L. 3 Biotech..

[44] Limmatvapirat C, Sirisopanaporn S, Kittakoop P (2004). Antitubercular and antiplasmodial constituents of *Abrus precatorius*. Planta Med.

[45] Muthaura CN, Keriko JM, Mutai C, Yenesew A, Gathirwa JW, Irungu BN, Nyangacha R, Mungai GM, Derese S (2015). Antiplasmodial potential of traditional antimalarial phytotherapy remedies used by the Kwale community of the Kenyan Coast. J Ethnopharmacol.

[46] Anywar GU, Kakudidi E, Oryem-Origa H, Schubert A, Jassoy C (2022). Cytotoxicity of medicinal plant species used by traditional healers in treating people suffering from HIV/AIDS in Uganda. Front Toxicol.

[47] Asase A, Akwetey GA, Achel DG (2010). Ethnopharmacological use of herbal remedies for the treatment of malaria in the Dangme West District of Ghana. J Ethnopharmacol.

[48] Bray DH, Warhurst DC, Connolly JD, O’neill MJ, Phillipson JD (1990). Plants as sources of antimalarial drugs. Part 7. Activity of some species of Meliaceae plants and their constituent limonoids. Phytother Res.

[49] Khalid SA (1989). Isolation and characterization of antimalarial agents of the neem tree *Azadirachta indica*. J Nat Prod.

[50] Kirira PG, Rukunga GM, Wanyonyi AW, Muregi FM, Gathirwa JW, Muthaura CN, Omar SA, Tolo F, Mungai GM, Ndiege IO (2006). Anti-plasmodial activity and toxicity of extracts of plants used in traditional malaria therapy in Meru and Kilifi Districts of Kenya. J Ethnopharmacol.

[51] Nanyingi MO, Kipsengeret KB, Wagate CG, Langat BK, Asaava LL, Midiwo JO, Midiwo JO, Clough J (2010). In vitro and in vivo antiplasmodial activity of Kenyan medicinal plants. Aspects of African Biodiversity Proceedings of the Pan-Africa Chemistry Network.

[52] Clarkson C, Maharaj VJ, Crouch NR, Grace OM, Pillay P, Matsabisa MG, Bhagwandin N, Smith PJ, Folb PI (2004). In vitro antiplasmodial activity of medicinal plants native to or naturalised in South Africa. J Ethnopharmacol.

[53] Frida L, Rakotonirina S, Rakotonirina A, Savineau JP (2007). In vivo and in vitro effects of *Bidens pilosa* L. (Asteraceae) leaf aqueous and ethanol extracts on primed-oestrogenized rat uterine muscle. Afr J Tradit Complement Altern Med.

[54] Lai BY, Chen TY, Huang SH, Kuo TF, Chang TH, Chiang CK, Yang MT, Chang CL (2015). *Bidens pilosa* formulation improves blood homeostasis and β-cell function in men: a pilot study. Evid Based Complement Alternat Med.

[55] Teng WC, Chan R, Suwanarusk W, Ong A, Ho HK, Russell B, Rénia L, Koh HL (2019). In vitro antimalarial evaluations and cytotoxicity investigations of *Carica papaya* leaves and carpaine. Nat Prod Comm.

[56] Melariri P, Campbell W, Etusim P, Smith P (2011). Antiplasmodial properties and bioassay-guided fractionation of ethyl acetate extracts from *Carica papaya* leaves. J Parasitol Res.

[57] Julianti T, De Mieri M, Zimmermann S, Ebrahimi SN, Kaiser M, Neuburger M, Raith M, Brun R, Hamburger M (2014). HPLC-based activity profiling for antiplasmodial compounds in the traditional Indonesian medicinal plant *Carica papaya* L. J Ethnopharmacol.

[58] Ndiege IO (2011). Anti-malarial activity and phytochemical studies of *Cissampelos mucronata* and *Stephania abyssinica*.

[59] Omole RA. Anti-malarial activity and phytochemical studies of *Cissampelos mucronata* and *Stephania abyssinica*. Department of Chemistry, Kenyatta University, Kenya. 2012

[60] Loomis TA, Hayes AW (1996). Loomis’s essentials of toxicology.

[61] Nwafor S, Akah P (1999). Studies on antiulcer properties of *C. mucronata* leaf extract India. J Exp Biol.

[62] Pascoe D (1983). Toxicology.

[63] Koch A, Tamez P, Pezzuto J, Soejarto D (2005). Evaluation of plants used for antimalarial treatment by the Maasai of Kenya. J Ethnopharmacol.

[64] Murugan K, Aarthi N, Kovendan K, Panneerselvam C, Chandramohan B, Kumar PM, Amerasan D, Paulpandi M, Chandirasekar R, Dinesh D, Suresh U, Subramaniam J, Higuchi A, Alarfaj AA, Nicoletti M, Mehlhorn H, Benelli G (2015). Mosquitocidal and antiplasmodial activity of *Senna occidentalis* (Cassiae) and *Ocimum basilicum* (Lamiaceae) from Maruthamalai hills against *Anopheles stephensi* and *Plasmodium falciparum*. Parasitol Res.

[65] Batista R, Silva Ade J, de Oliveira AB (2009). Plant-derived antimalarial agents: new leads and efficient phytomedicines. Part II. Non-alkaloidal natural products. Molecules.

[66] Lusakibanza M, Mesia G, Tona G, Karemere S, Lukuka A, Tits M, Angenot L, Frédérich M (2010). In vitro and in vivo antimalarial and cytotoxic activity of five plants used in Congolese traditional medicine. J Ethnopharmacol.

[67] Adia MM, Emami SN, Byamukama R, Faye I, Borg-Karlson AK (2016). Antiplasmodial activity and phytochemical analysis of extracts from selected Ugandan medicinal plants. J Ethnopharmacol.

[68] Froelich S, Onegi B, Kakooko A, Siems K, Schubert C, Jenett-Siems K (2007). Plants traditionally used against malaria: phytochemical and pharmacological investigation of *Momordica foetida*. Rev bras farmacogn.

[69] Obbo CJD, Kariuki ST, Gathirwa JW, Olaho-Mukani W, Cheplogoi PK, Mwangi EM (2019). In vitro antiplasmodial, antitrypanosomal and antileishmanial activities of selected medicinal plants from Ugandan flora: refocusing into multi-component potentials. J Ethnopharmacol.

[70] Tanko Y, Yaro AH, Isa AI, Yerima M, Saleh MIA, Mohammed A (2007). Toxicological and hypoglycaemic studies on the leaves of *Cissampelos mucronata* (Menispermaceae) on blood glucose levels of streptozotocin-induced diabetic Wistar rats. J Med Plant Res.

[71] Fandohan P, Gnonlonfin B, Laleye A, Gbenou JD, Darboux R, Moudachirou M (2008). Toxicity and gastric tolerance of essential oils from *Cymbopogon citratus*, *Ocimum gratissimum* and *Ocimum basilicum* in Wistar rats. Food Chem Toxicol.

[72] Kayembe JS, Taba KM, Ntumba K, Tshiongo MTC, Kazadi TK (2010). *In vitro* antimalarial activity of 20 quinones isolated from four plants used by traditional healers in the Democratic Republic of Congo. J Med Plants Res.

[73] Tona L, Mesia K, Ngimbi NP, Chrimwami B, Okondahoka CK, de Bruyne T, Apers S, Hermans N, Totte J, Pieters L, Vlietinck AJ (2001). In-vivo antimalarial activity of *Cassia occidentalis*, *Morinda morindoides* and *Phyllanthus niruri*. Ann Trop Med Parasitol.

[74] Silva MG, Aragão TP, Vasconcelos CF, Ferreira PA, Andrade BA, Costa IM, Costa-Silva JH, Wanderley AG, Lafayette SS (2011). Acute and subacute toxicity of *Cassia occidentalis* L. stem and leaf in Wistar rats. J Ethnopharmacol.

[75] Murithi C, Fidahusein D, Nguta J, Lukhoba C (2014). Antimalarial activity and in vivo toxicity of selected medicinal plants naturalized in Kenya. Int J Educ Res.

[76] Nguta JM, Mbaria JM (2013). Brine shrimp toxicity and antimalarial activity of some plants traditionally used in treatment of malaria in Msambweni district of Kenya. J Ethnopharmacol.

[77] Martinello F, Soares SM, Franco JJ, Santos AC, Sugohara A, Garcia SB, Curti C, Uyemura SA (2006). Hypolipemic and antioxidant activities from *Tamarindus indica* L. pulp fruit extract in hypercholesterolemic hamsters. Food Chem Toxicol.

[78] Orwa JA, Ngeny L, Mwikwabe NM, Ondicho J, Jondiko IJ (2013). Antimalarial and safety evaluation of extracts from *Toddalia asiatica* (L) Lam. (Rutaceae). J Ethnopharmacol.

[79] Onguén P, Ntie-Kang F, Lifongo LL, Ndom JC, Sippl W, Mbaze LM (2013). The potential of anti-malarial compounds derived from African medicinal plants, part I: a pharmacological evaluation of alkaloids and terpenoids. Malar J.

[80] Stangeland T, Alele PE, Katuura E, Lye KA (2011). Plants used to treat malaria in Nyakayojo sub-county, western Uganda. J Ethnopharmacol.

[81] Omoregie ES, Pal A, Sisodia B (2011). In vitro antimalarial and cytotoxic activities of leaf extracts of *Vernonia amygdalina* (Del.). Nigerian J Basic Appl Sci..

[82] Lacroix D, Prado S, Kamoga D, Kasenene J, Namukobe J, Krief S, Dumontet V, Mouray E, Bodo B, Brunois F (2011). Antiplasmodial and cytotoxic activities of medicinal plants traditionally used in the village of Kiohima. Uganda J Ethnopharmacol.

[83] Challand S, Willcox M (2009). A clinical trial of the traditional medicine *Vernonia amygdalina* in the treatment of uncomplicated malaria. J Alternat Compl Med.

[84] Wube A, Bucar F, Gibbons S, Asres K, Rattray L, Croft SL (2008). Anti-protozoal activity of sesquiterpenes from *Warburgia ugandensis* towards *Trypanosoma bruceirhodesiense* and *Plasmodium falciparum* in vitro. Planta Med.

[85] Were PS, Kinyanjui P, Gicheru MM, Mwangi E, Ozwara HS (2010). Prophylactic and curative activities of extracts from *Warburgia ugandensis* Sprague (Canellaceae) and *Zanthoxylum usambarense* (Engl) Kokwaro (Rutaceae) against *Plasmodium knowlesi* and *Plasmodium berghei*. J Ethnopharmacol.

[86] Okello D, Komakech R, Matsabisa MG, Kang YM (2018). A review on the botanical aspects, phytochemical contents and pharmacological activities of *Warburgia ugandensis*. J Med Plants Res.

