# Supplementary material for: Medicinal plants used for treatment of malaria by indigenous communities of Tororo District, Eastern Uganda
Source: Trop Med Health. 2023 Jun 12;51:34. doi: 10.1186/s41182-023-00526-8 (PMC10258082; doi:10.1186/s41182-023-00526-8)
Supplement: Supplementary file 1 — Additional file 1. S1. Questionnaire used in the ethnobotanical survey of medicinal plants used for treatment of malaria by indigenous communities of Tororo District, Eastern Uganda. [file 41182_2023_526_MOESM1_ESM.docx]

**ETHOBOTANICAL DATA COLLECTION TOOL**

**Project title: Development of a Safe and Efficacious Anti-malarial Drug from Traditional medicine (DESAT)**

**Introduction**

My name is _________________, I am from Makerere University. We are doing research to determine herbal medicines which are effective for the treatment of malaria. I would like to invite you to participate in an interview as part of this project. If you choose to take part, we will not use your real name, and what you tell me will only be used for this research. This interview will take around 40 - 60 minutes – Would you like to take part?

Do you have any questions? Please sign if you agree to take part_______________________

**Part 1: Socio-demographics characteristics of the respondents**

1. Age ………………………… years
2. Gender: Female Male
3. Education; Primary Secondary Tertiary University

1. Ethnicity: Japhadhola Itesot other
2. Place/ Village …………………. Sub county ……………………..District ……………………………
3. Marital status: Married Single Widow
4. Religion: Catholic Anglican Pentecostal Muslim None
5. Occupation: Formal employment Subsistence farmer casual labour others
6. Traditional medicine practitioner (Herbalist): Yes No

**Part 2: Knowledge about malaria and its management**

1. Have you or any of your family members suffered from malaria in the last six month?

| Yes |  | No |  |
| --- | --- | --- | --- |

1. Which of these signs did you have or your family member had? Tick all that applies
2. High temperature
3. Weakness
4. Headache
5. Diarrhoea
6. Convulsions
7. Others (specify) ______________________
8. When you suffer from malaria, how do you treat yourself/family members first?
9. TM alone
10. Modern medicine alone
11. Both
12. When you do not recover the first time how do you treat yourself/family member?
13. TM alone
14. Modern medicine alone
15. Both
16. Do you use this treatment alone or in combination with modern medicine?
17. TM alone
18. Modern medicine alone
19. Both TM and modern medicine
20. When you use TM, what material do you use?
21. Plants
22. Animals
23. Inorganic material
24. Incantations
25. How did you learn about these TM treatments?
26. Media Radio
27. Media TV
28. Relative (please specify e.g. uncle)
29. Parents (please specify e.g. father)
30. Others (please specify)

**Part 3: Knowledge and practices about medicinal plants for malaria**

1. Please tell me the plants that you use to treat malaria. (Please enter in the table below).

- *Plant form include tree, herb, shrub, climber, grass*
- *Plant parts include flowers, seed, fruit, leaves, stem bark, stem wood, root bark, root wood, resin, hanging roots (e.g. mutuba)*
- Please tell me how you process these plants. (*I cook the plant = decoction; I squeeze in water = maceration; I put in hot water = infusion; I process a powder and add to hot water (****pi****); I process powder and boil (****pd****);.* (Please enter in the table below)

| **Plant (local name)** | **Plant form / habit** | **Part** | **Mode of preparation** |
| --- | --- | --- | --- |
|  |  |  |  |
|  |  |  |  |
|  |  |  |  |

- Enter separately for separate formulas if the respondent mentions more than one.

1. Tell me how you administer to the patient. (Please specify the quantities administered and the frequency in the table below).

- Administration: (Oral, *steam bath; smoking; crush and bathe).*
- *Dose: Move with a spoon and a cup for estimation amounts.*
- *Frequency: Once, twice or thrice a day for one, two or three weeks*
- *Side effects: Vomiting, diarrhoea, sedation, Urination, dizziness, rash, change of urine colour, loss of appetite*
- *Packaging materials: Bottles, jerricans, polyethene bags, newspaper*

| **Route of administration** | **Dose** | **Frequency and duration** | **Side effects** | **Packaging** |
| --- | --- | --- | --- | --- |
|  |  |  |  |  |
|  |  |  |  |  |
|  |  |  |  |  |

1. Where do you collect the plants from?
2. Garden
3. Compound
4. Wild
5. Other
6. Do you think TM for treating malaria works? (Please tick what applies).

| Very effective |  | Effective |  | Somewhat effective |  | Not effective |  |
| --- | --- | --- | --- | --- | --- | --- | --- |

1. Do you store the traditional medicine? For how long do you store the traditional medicine?
2. How do you preserve the medicine for storage?

**Part 4: Interview guide for the focus group discussions**

1. What causes malaria and is it transmitted?
2. Which group of people are mostly affected by malaria?
3. How do you tell that someone has malaria?
4. How do you manage malaria in your community?
5. What challenges exist for using traditional medicine to treat malaria?
6. What challenges exist for using modern medicine to treat malaria?
7. Where do you get the plant species for preparation of herbal medicines for malaria?
8. Do you grow some of these traditional medicines or you buy them?

For more details about the study, please contact Prof. John R.S. Tabuti on Tel: +256772960880 or Email: [jtabuti@gmail.com](mailto:jtabuti@gmail.com)

**END**
